# Myopericarditis and Pulmonary Edema

**DOI:** 10.21980/J8.52187

**Published:** 2025-10-31

**Authors:** Lubna Ahmad Saffarini

**Affiliations:** * Dubai Academic Health Corporation, Rashid Hospital, Department of Emergency Medicine, Dubai; ^ Institute of Learning, Mohammed Bin Rashid University of Medicine and Health Sciences, Dubai

## Abstract

**Audience:**

This oral board case is intended to be used with senior emergency medicine residents.

**Introduction:**

Pericarditis and myocarditis are two disease entities that refer to inflammation of the pericardium and the myocardium.^1^ In clinical practice, when they occur together the term myopericarditis is used.^1^ Myopericarditis is a disease etiology that is uncommonly encountered in the emergency department (ED). Pericarditis accounts for 0.2% of all cardiovascular admissions and myopericarditis is even more rare with an unknown exact incidence.^2^ These pathologies can present with a wide spectrum of complaints varying from a simple case of chest pain to more severe hypotensive pulmonary edema, cardiogenic shock, and cardiac arrest.^2^ Since these pathologies primarily affect the younger patient population,^2^ they require careful consideration by the Emergency Physician (EP) as well as a systematic comprehensive approach to managing these critically ill patients. The majority of cases are of idiopathic origin; however, the causes of pericarditis are divided into infectious and noninfectious, with infectious cases primarily resulting from viral infections.^3^ Causes of myocarditis, on the other hand, can be divided into infectious, immune-mediated, and toxic.^2^ Although myopericarditis is a rare disease, its high acuity makes it an important case to enhance the educational experience of emergency medicine residents and their associated faculty.

**Educational Objectives:**

At the end of this oral board session, learners will be able to: 1) Demonstrate the ability to evaluate and treat a somnolent and hypoxic patient, 2) Identify a critical airway situation and manage it with a holistic approach, 3) Interpret the history, physical examination, ECG, and chest x-ray findings and discuss the list of differential diagnoses, 4) Identify a state of cardiogenic shock induced by myopericarditis and treat it appropriately, 5) Assess the presence of pericardial effusion and cardiac tamponade utilizing bedside echocardiography.

**Educational Methods:**

This is an oral board case and is implemented in a face-to-face setup or virtually on multiple available technological modalities.

**Research Methods:**

This oral mock code was developed for senior emergency medicine residents to prepare for their oral board exams. Each session lasted approximately 25 minutes, with 10 minutes for the case and 15 minutes for debriefing. The case, initially designed as myopericarditis-induced pulmonary edema progressing to shock and rhabdomyolysis, was later simplified because it was challenging for residents to identify all the elements within the time allotted. Positive feedback indicated the case was educational and beneficial, though extending the case duration to 15 minutes was recommended. Performance was assessed using the Accreditation Council for Graduate Medical Education (ACGME) core competencies with a scoring scale of 1 – 8, with 1 – 4 being unacceptable performance and 5 – 8 being acceptable. Efficacy was determined by case completion and participation in structured debriefing. Immediate verbal feedback and a post-session Likert-scale survey further supported its educational value. The case is also adaptable for simulation-based group learning.

**Results:**

Five senior residents (three PGY4 and two PGY3) completed the oral case, achieving an average score of 6.01/8, with only one resident completing all critical actions. The most commonly missed intervention was administering aspirin or ibuprofen, and none considered advanced circulatory support despite persistent hypotension. The case was rated highly for educational value (5/5), all of them reported that it increased their medical knowledge, and it was similar to real-life scenarios. Some also noted it increased their confidence level and the case difficulty was rated as moderate (3/5) by the majority of the residents.

**Discussion:**

The educational content of this case is effective because it is multifaceted and requires consideration of multiple factors during both the approach and management. These elements produce an excellent case for discussion, practice, and examination. Points learned while implementing the case were that, 1) its degree of difficulty is more suitable for senior rather than junior learners because it involves a critically ill patient with multiple simultaneous issues to be managed, 2) it requires a well-rounded and systematic approach to achieve all critical actions, and 3) the appropriate prompts should be utilized to cover all aspects in the allotted 15 minutes for the case.

**Topics:**

Myopericarditis, pulmonary edema, cardiogenic shock, sepsis, septic shock.

## USER GUIDE

**Table:** 

List of Resources:	
Abstract	1
User Guide	3
For Examiner Only	6
Oral Boards Assessment	12
Stimulus	15
Debriefing and Evaluation Pearls	29

**Learner Audience:**

Senior Residents

**Time Required for Implementation:**

Case: 15 minutes

Debriefing: 10 minutes

**Recommended number of learners per instructor:**

One learner

**Topics:**

Myopericarditis, pulmonary edema, cardiogenic shock, sepsis, septic shock.

**Objectives:**

At the end of this oral board session, learners will be able to:

1. Demonstrate the ability to evaluate and treat a somnolent and hypoxic patient
2. Identify a critical airway situation and manage it with a holistic approach
3. Interpret the history, physical examination, ECG, and chest x-ray findings and discuss the list of differential diagnoses
4. Identify a state of cardiogenic shock and treat it appropriately
5. Assess the presence of pericardial effusion and cardiac tamponade utilizing bedside echocardiography

### Linked objectives, methods and results

The case presented allows learners to have experience within a safe environment in assessing and managing multiple critical scenarios such as the hypoxic patient with a critical airway (objectives 1 – 2) and myopericarditis-induced pulmonary edema worsening into shock (objectives 4 – 5). It will allow learners to have a systematic approach (objective 1), to review the list of differential diagnoses (objective 3), and to appropriately diagnose and manage the case (objectives 1 – 5). This format was chosen because of its ease of use and implementation. Also, a discussion at the end of the scenario with debriefing will help consolidate the educational points learned.

### Results and tips for successful implementation

The case was used as an oral mock code for senior emergency medicine residents as practice for their oral board exam. Each case round took approximately 25 minutes and was divided as follows: each resident had 10 minutes for the case and 15 minutes of debriefing and discussion. Positive feedback was provided from the residents, and they found the case useful and educational. Initially, the case was presented as myopericarditis-induced pulmonary edema progressing into shock and associated rhabdomyolysis. However, after implementing it, it was clear that the scenario was already multipronged and difficult, and most residents didn’t conclude that the patient also developed rhabdomyolysis. It was also noted that 10 minutes is not enough to conclude most of the elements of the case, so a duration of 15 minutes is suggested. The case can also be implemented in a simulation-based training session in a group setting. It is advisable that reading materials and references be given to the learner towards the end of the debriefing and feedback session.

The learners participating in the oral board case provided immediate verbal feedback after the debriefing and review session. A short anonymous online survey was also distributed to them on Google Forms to assess their educational experience using a Likert-Scale question format. The examiner assessed and scored resident performance based on the core competencies of the Accreditation Council for Graduate Medical Education (ACGME). The scoring scale used ranges from 1 – 8, with 1 – 4 being unacceptable performance and 5 – 8 being acceptable. Efficacy was determined by the resident’s full completion of the oral board case and participation in a debriefing session where key educational concepts were discussed and reviewed. Three PGY4 and two PGY3 residents completed the oral case, thus a total of five residents. The average score was 6.01/8, with only one resident completing all critical actions. The most commonly missed critical action was administering aspirin or ibuprofen. Two residents gave the patient fluid boluses despite signs and symptoms of fluid overload and none of them considered extracorporeal membrane oxygenation (ECMO), intra-aortic balloon pump (IABP), or left ventricular assist device (LVAD) in the management despite persistent hypotension. The rated educational value of the case by the residents was 5/5, and all reported that it increased their medical knowledge. Two of them reported that it increased their confidence level in managing a similar case in the future. The case was given a score of 3/5 when rating its difficulty by four residents and one gave it a score of 2/5. All of them agreed that the case was similar to real-life scenarios.

### Pearls

Myocarditis and pericarditis are difficult conditions to diagnose in the emergency department (ED) since their presenting complaints are commonly encountered across other disease entities.^2^ They commonly present with chest pain, shortness of breath, and palpitations in otherwise healthy young individuals, often occurring within weeks of a preceding viral infection.^2^ Acute pericarditis is diagnosed when at least two of the following criteria are present: pleuritic or pericarditic chest pain, a pericardial friction rub on physical examination, electrocardiographic changes such as diffuse concave ST-segment elevation with PR-segment depression, or the presence of a pericardial effusion.^1^ Myopericarditis is identified when these findings are accompanied by at least one additional feature, such as elevated cardiac biomarkers, new-onset left ventricular systolic dysfunction on echocardiography or cardiac magnetic resonance imaging (CMR), or evidence of myocardial inflammation on CMR.^1^

The European Society of Cardiology notes that patients with biopsy-confirmed inflammatory heart disease can present with symptoms of acute coronary syndrome (ACS), heart failure, cardiogenic shock, and unstable dysrhythmias.^2^ The patient in this case presented with pulmonary edema and shortness of breath that progressed to cardiogenic shock. In the ED, the initial treatment of pulmonary edema is guided by the patient’s complaints and hemodynamic status. In a normotensive, hypoxic, and tachypneic patient with heart failure, the mainstay of therapy includes non-invasive ventilation (NIV) to promote alveolar clearance, and loop diuretics to promote diuresis.^3^,^5^ High-flow nasal cannula (HFNC) may also be used at flow rates of 40–50 liters per minute; however, it does not generate sufficient positive end-expiratory pressure (PEEP) to adequately promote alveolar fluid clearance.^5^ Also, its use in the context of heart failure has not been adequately studied, and therefore, no strong recommendation can be made regarding its efficacy in this setting.^5^ Otherwise, if NIV is contraindicated or has failed, then endotracheal intubation is indicated.^5^

Acute heart failure and cardiogenic shock should be managed in the standard manner, without disease-specific therapy for myocarditis.^2^ For patients in cardiogenic shock, norepinephrine combined with an inotropic agent such as dobutamine or milrinone is preferred over epinephrine alone.^2^ However, when dobutamine is used alone in cases where the systolic blood pressure is less than or equal to 90mmHg, it may lead to vasodilation and hypotension.^4^ In cases of persistent hypotension or when the patient is in extremis and requires mechanical ventilation, push-dose pressors can be used to increase the blood pressure.^7^,^8^ Epinephrine is a commonly used pressor as push dose because it exhibits both alpha-1 and alpha-2 adrenergic effects as well as beta-1 and beta-2 activity, making it effective.^7^,^8^ Its onset of action occurs in less than one minute, and although a single dose may last up to ten minutes, the hemodynamic effects typically resolve within five minutes. To prepare the push-dose epinephrine, a 10 mL syringe is filled with 9 mL of normal saline and then 1 mL of epinephrine from the cardiac ampule (which contains 10 mL of epinephrine at a concentration of 100 mcg/mL, or 1:10,000) is added into the syringe. The final concentration of epinephrine in the syringe is 10 mcg/mL (1:100,000).^7^,^8^

The mainstay of treatment for pericarditis regardless of the cause is anti-inflammatory drugs combined with supportive care.^2^ The ESC (European Society of Cardiology) guidelines recommend non-steroidal anti-inflammatory drugs (NSAIDs) such as aspirin (650–1000 mg orally every 8 hr) and ibuprofen (600 mg orally every 8 hr) as the first-line therapy in cases of normal renal function.^2^ If there is a contraindication to NSAIDs or failure of treatment, then corticosteroids can be used with a taper regimen.^2^ Colchicine is another first-line therapy recommended to be added to NSAIDs or corticosteroids in acute pericarditis.^1^,^2^ Myocarditis management, though, focuses on supportive care by managing resultant cardiac complications.^2^

Even though the gold-standard test, an endomyocardial biopsy, is not available in the ED, the evaluation of a patient suspected to have myocarditis should include an ECG, troponin, and inflammatory markers, though normal results do not exclude the diagnosis.^1^,^2^ A bedside point-of-care ultrasound (POCUS) should be utilized to detect effusion even though it often reveals normal cardiac function.^1^ While increased pericardial echogenicity has been suggested as a marker of inflammation, it remains a non-specific and limited finding of pericarditis.^1^ In some cases, patients may develop a significant pericardial effusion, which may or may not result in hemodynamic compromise due to tamponade.^1^ Another utility for the bedside ultrasound is to assess volume status in patients with shock.

Patients experiencing refractory cardiogenic shock with hemodynamic instability may require mechanical circulatory support.^2^ Modalities such as intra-aortic balloon pump (IABP), ventricular assist devices (VADs), or extracorporeal membrane oxygenation (ECMO) can serve as a bridge to either ventricular function recovery or cardiac transplantation.^2^ Early initiation within the first 24 hours has been associated with improved outcomes in acute decompensated myocarditis.^2^

## Figures and Tables

**Table:** 

**Vitals:**	HR 120	BP 110/70	RR 20	Temp 40°C	SPO2 80% on 15L NRB

**Table:** 

	Milestone	Did not achieve level 1	Level 1	Level 2	Level 3
**1**	**Emergency Stabilization (PC1)**	□ **Did not achieve Level 1**	□ **Recognizes abnormal vital signs**	□ **Recognizes an unstable patient, requiring intervention** **Performs primary assessment** **Discerns data to formulate a diagnostic impression/plan**	□ **Manages and prioritizes critical actions in a critically ill patient** **Reassesses after implementing a stabilizing intervention**
**2**	**Performance of focused history and physical (PC2)**	□ **Did not achieve Level 1**	□ **Performs a reliable, comprehensive history and physical exam**	□ **Performs and communicates a focused history and physical exam based on chief complaint and urgent issues**	□ **Prioritizes essential components of history and physical exam given dynamic circumstances**
**3**	**Diagnostic studies (PC3)**	□ **Did not achieve Level 1**	□ **Determines the necessity of diagnostic studies**	□ **Orders appropriate diagnostic studies** **Performs appropriate bedside diagnostic studies/procedures**	□ **Prioritizes essential testing** **Interprets results of diagnostic studies** **Considers risks, benefits, contraindications, and alternatives to a diagnostic study or procedure**
**4**	**Diagnosis (PC4)**	□ **Did not achieve Level 1**	□ **Considers a list of potential diagnoses**	□ **Considers an appropriate list of potential diagnosis** **May or may not make correct diagnosis**	□ **Makes the appropriate diagnosis** **Considers other potential diagnoses, avoiding premature closure**
**5**	**Pharmacotherapy (PC5)**	□ **Did not achieve Level 1**	□ **Asks patient for drug allergies**	□ **Selects an appropriate medication for therapeutic intervention, considering potential adverse effects**	□ **Selects the most appropriate medication(s) and understands mechanism of action, effect, and potential side effects** **Considers and recognizes drug-drug interactions**
**6**	**Observation and reassessment (PC6)**	□ **Did not achieve Level 1**	□ **Reevaluates patient at least one time during the case**	□ **Reevaluates patient after most therapeutic interventions**	□ **Consistently evaluates the effectiveness of therapies at appropriate intervals**
**7**	**Disposition (PC7)**	□ **Did not achieve Level 1**	□ **Appropriately selects whether to admit or discharge the patient**	□ **Appropriately selects whether to admit or discharge** **Involves the expertise of some of the appropriate specialists**	□ **Educates the patient appropriately about their disposition** **Assigns patient to an appropriate level of care (ICU/Tele/Floor)** **Involves expertise of all appropriate specialists**
**22**	**Patient centered communication (ICS1)**	□ **Did not achieve level 1**	□ **Establishes rapport and demonstrates empathy to patient (and family)** **Listens effectively**	□ **Elicits patient’s reason for seeking health care**	□ **Manages patient expectations in a manner that minimizes potential for stress, conflict, and misunderstanding.**
**23**	**Team management (ICS2)**	□ **Did not achieve level 1**	□ **Recognizes other members of the patient care team during case (nurse, techs)**	□ **Communicates pertinent information to other healthcare colleagues**	□ **Communicates a clear, succinct, and appropriate handoff with specialists and other colleagues** **Communicates effectively with ancillary staff**

**Table:** 

**#1**	**Patient Information**
**#2**	**Arterial Blood Gas**
**#3**	**FBC**
**#4**	**Urea, Electrolytes, Glucose, and Creatinine**
**#5**	**Troponin and Cardiac pro-BNP**
**#6**	**Coagulation Profile**
**#7**	**Septic Workup**
**#8**	**Urinalysis**
**#9**	**ECG and Rhythm Strip**
**#10**	**Portable Chest X-ray**
**#11**	**Pulmonary Edema – B Lines**
**#12**	**Pericardial Effusion – No Tamponade**
**#13**	**IVC - Plethoric**

**Table:** 

**Patient’s Name:**	**M.A.**
**Age:**	**28-years-old**
**Gender:**	**Male**
**Chief Complaint:**	**Fever, shortness of breath, and somnolence**
**Person Providing History: paramedics and roommate**	
**Vital Signs:**	
**Temperature:**	**40°C**
**Blood Pressure:**	**110/70 mmHg**
**Heart Rate:**	**120 beats per minute**
**Respiratory Rate:**	**20 breaths per minute**
**Pulse Oximetry:**	**80% on 15L NRB**
**Weight:**	**65 kilograms**

**Table:** 

pH	7.413
pCO2	26.3 mmHg
pO2	62.1 mmHg
HCO3	16.8 mmol/L
O2 saturation	80%

**Table:** 

WBC	19.1 × 10^3^/μL
Hgb	16.3 g/dL
Hct	48.3%
Platelets	176 × 10^3^/μL
Differential	
Neutrophils	91.5%
Lymphocytes	4.9%
Monocytes	3.5%
Eosinophils	0%

**Table:** 

Na	134 mEq/L
K	3.9 mEq/L
Cl	98 mEq/L
BUN	87 mg/dL
Cr	2.27 mg/dL
Glucose	128 mg/dL

**Table:** 

Troponin	17,021 ng/L
Pro-BNP	3,988 pg/mL

**Table:** 

PT	11.2 seconds
PTT	28.2 seconds
INR	1.04
Dimer	500 ng/mL

**Table:** 

CRP	167 mg/L
Procalcitonin	1.2 ng/mL
Lactate	3 mmol/L
Blood culture	Pending

**Table:** 

Appearance	Hazy
Color	Yellow
Glucose	Negative
Ketones	Negative
Specific Gravity	1.024
Blood	1+
pH	5.5
Protein	1+
Nitrite	Negative
Leukocyte	Negative
WBC	5–10/HPF
RBC	25–30/HPF
Squamous Cells	Occasional

**Figure:**
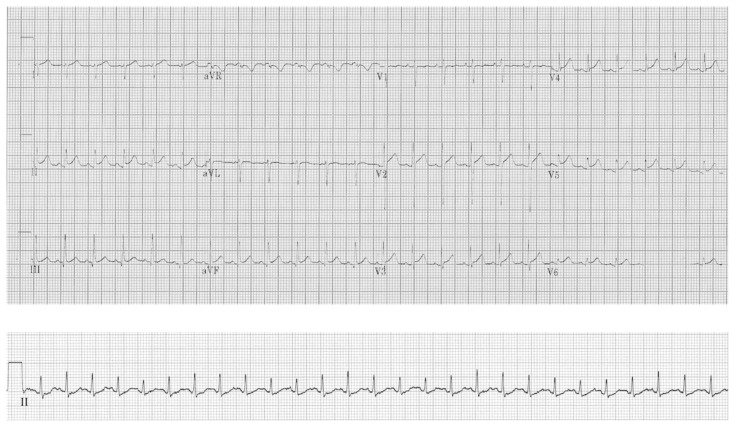


**Figure:**
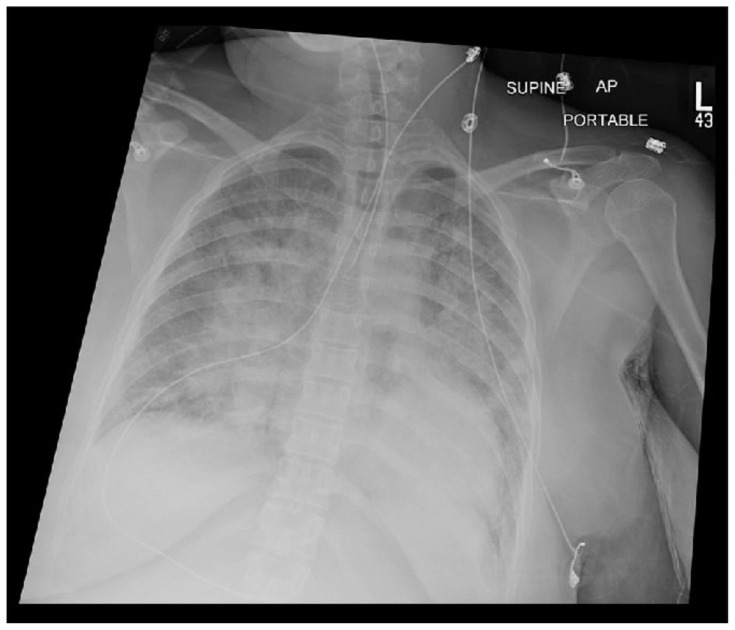


**Figure:**
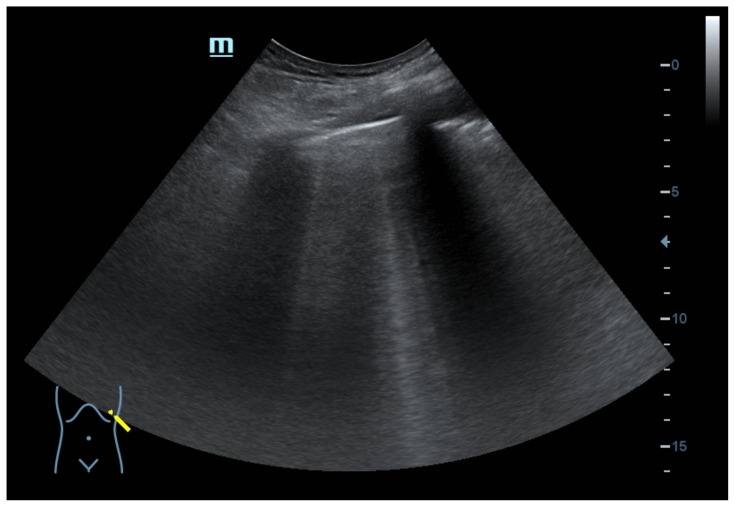


**Figure:**
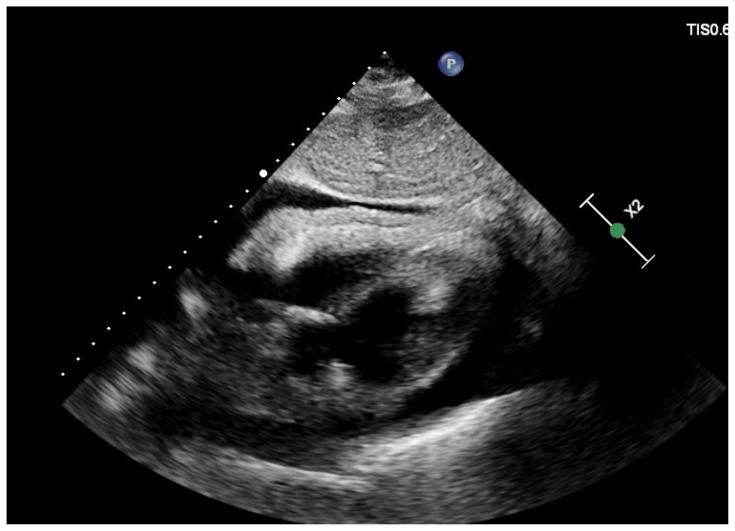


**Figure:**
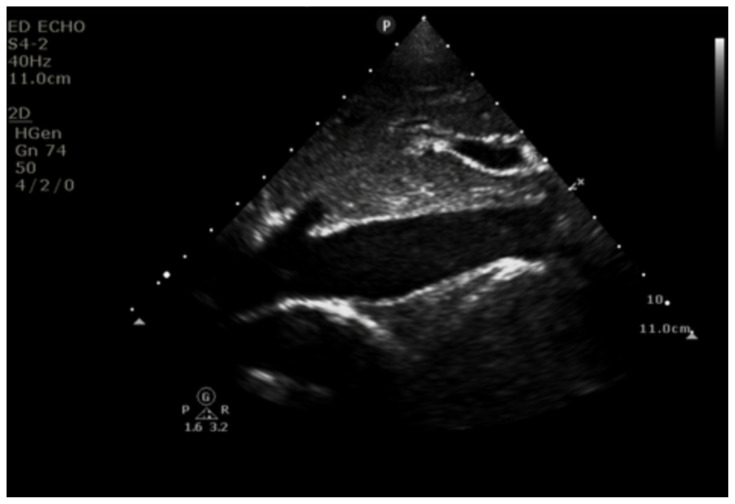


## FOR EXAMINER ONLY

### Oral Case Summary

**Diagnosis: Myopericarditis and Pulmonary Edema**

**Case Summary:** The patient is a 28-year-old male, M.A., brought by the paramedics with a history of fever, shortness of breath, and somnolence. His symptoms started five days ago and worsened today. The patient is previously healthy and lives in his work accommodation with his roommate who had a recent flu-like illness. The roommate called the ambulance today when he noted M.A has breathing difficulty and is becoming somnolent.

**Order of Case:** The patient arrives on a gurney in the head-up sitting position, is attached to a 15L non-rebreather mask (NRB), and has one IV line inserted in the right antecubital fossa by the paramedics. The learner should ask for the full set of vitals with the initial ABCs and should recognize that the patient has a saturation of 80% on 15L NRB, a respiratory rate (RR) of 20 breaths per minute (brpm), a blood pressure (BP) of 110/70, a pulse of 120 beats per minute (bpm), a Temperature of 40°C, is speaking in interrupted sentences with no stridor, has bilateral crepitations up to the apices of the lungs, and has cold and clammy skin with equal pulses. The learner should immediately request to attach the patient to a cardiac monitor with pads, order a second IV line, attach the patient to a non-invasive ventilation (NIV) machine, titrate the FiO2 and PEEP ( positive end-expiratory pressure), prepare for possible intubation, and ask for backup. The learner should also order a stat random blood sugar (RBS) for somnolence.

The learner should further examine the patient and recognize that he is somnolent but arousable with a GCS (Glasgow Coma Scale) of 15, has bilateral lower limbs pitting edema, and hepatomegaly. He does not have any rash on exposure or signs of trauma and doesn’t exhibit any meningeal signs. The learner should order furosemide 40mg IV to promote diuresis. The appropriate blood tests should also be ordered including full blood count (FBC), urea and electrolytes (U&E), creatinine, troponin, cardiac pro-B-type natriuretic peptide (pro-BNP), dimer, prothrombin time (PT), partial thromboplastin time (PTT), c-reactive protein (CRP), procalcitonin, blood culture, urine routine and culture, lactic acid, and a respiratory screening panel. Learners should also order an ECG, a blood gas, a chest x-ray, give a broad-spectrum antibiotic for suspicion of sepsis, and prepare for a norepinephrine infusion. A follow-up of the ECG and chest x-ray should be done immediately; learners should recognize the abnormal findings and request a bedside ultrasound to assess the presence of pericardial effusion on echocardiography, check for B-lines, and assess volume status by scanning the inferior vena cava (IVC). The learner should also order aspirin orally 650mg or ibuprofen 800mg after finding the pericardial effusion for suspicion of myopericarditis.

After the initial assessment of ABCs and a secondary survey, the learner should reassess the patient and recognize that his hypoxia is not improving with an SPO2 of 88%, he is becoming more tachypneic with an RR of 30 brpm, and his BP is 80/40 with a MAP (mean arterial pressure) of 53. The learner should stop the NIV, attach the patient to a high flow nasal cannula (HFNC) machine, start a norepinephrine infusion with a target MAP of 65 – 80, and repeat the bedside echocardiography to rule out a tamponade. After the norepinephrine infusion is started, the BP increases to 82/50 with a MAP of 61, the patient is noted to become more somnolent, and his GCS drops to 10/15. A decision to intubate should be taken for persistent hypoxia and drop in GCS. The learner should increase the norepinephrine infusion and start the patient on a push dose pressor in anticipation of further deterioration during intubation. After a successful intubation using the appropriate medications, the learner should request to attach the patient to the mechanical ventilator, start the patient on sedation, order an ECG, repeat vitals, central line insertion, arterial line insertion, foley catheter insertion, and a repeat chest x-ray.

The learner then consults the intensive care unit (ICU) and cardiology teams for a possible extracorporeal membrane oxygenation (ECMO), intra-aortic balloon pump (IABP), or left ventricular assist device (LVAD), and is admitted to the ICU.

**Disposition:** Admission to the ICU

**Critical Actions:**

1. Recognizes a normotensive hypoxic pulmonary edema and connects the patient to an NIV machine
2. Performs a bedside echocardiography and assesses for pericardial effusion and cardiac tamponade
3. Administers aspirin or ibuprofen (NSAID)
4. Starts peripheral norepinephrine when the patient becomes hypotensive
5. Intubates the patient due to persistent hypoxia and drop in GCS but optimizes norepinephrine infusion and considers using push dose pressors

### Historical Information

**Chief Complaint:** Fever, Shortness of breath, and Somnolence

**History of Present Illness:** The patient is a 28-year-old male, M.A., brought by the paramedics with a history of fever, shortness of breath, and somnolence. His symptoms started five days ago and worsened today. The patient is previously healthy and lives in his work accommodation with his roommate who had a recent flu-like illness. The roommate called the ambulance today when he noted M.A has breathing difficulty and is becoming somnolent.

**Past Medical History:** None

**Past Surgical History:** None

**Patient’s Medications and Supplements:** None

**Allergies:** None

**Social history:**

- Smoking: None
- Tobacco: None
- Drug use: None

**Family history:** None

### Physical Exam Information

**Weight:** 65 kg

**General appearance:** somnolent and tachypneic

**Primary survey:**

- **Airway:** patent, speaks in full sentences
- **Breathing:** equal air entry bilaterally, bilateral crepitations up to the apices of the lungs
- **Circulation:** equal pulses bilaterally, cold and clammy skin

**Physical examination:**

**General appearance:**

- **HEENT:**
  ○ **Head:** no signs of trauma
  ○ **Eyes:** pupils are equal and reactive bilaterally
  ○ **Ears:** normal examination
  ○ **Nose:** nasal flaring
  ○ **Oropharynx/Throat:** dry mucus membranes
- **Neck:** no raised JVD (jugular vein distention)
- **Chest:** equal air entry bilaterally, bilateral crepitations up to the apices of the lungs
- **Cardiovascular:** pericardial friction rub and positive S3 gallop murmur
- **Abdominal/GI:** hepatomegaly, abdomen is soft, with no tenderness or guarding
- **Genitourinary:** within normal limits
- **Rectal:** within normal limits
- **Extremities:** bilateral lower limb pitting edema
- **Back:** within normal limits
- **Neuro:** GCS 15, moves all limbs, no neurologic deficit
- **Skin:** cold and clammy, no rash, no signs of trauma
- **Lymph:** within normal limits
- **Psych:** unable to assess

### Critical Actions and Cueing Guidelines

1. **Critical Action 1: Recognizes a normotensive hypoxic pulmonary edema and connects the patient to an NIV machine**
  The critical action is met by the learner ordering to attach the patient to an NIV machine immediately
  Cueing Guideline:
  *If the learner doesn’t order for an NIV machine to be connected to the patient, the nurse should say, “The patient’s saturation is dropping to 78% on a NRB mask with 15L; should we do something?”*
  *If the learner decides to connect the patient to an HFNC machine, the hypoxia will mildly improve to 82%*
2. **Critical Action 2: Performs a bedside echocardiography and assesses for pericardial effusion and cardiac tamponade**
  The critical action is met when the learner performs the bedside echocardiography to assess for a pericardial effusion and a tamponade when the patient becomes hypotensive
3. **Critical Action 3: Administers aspirin or ibuprofen (NSAID)**
  The critical action is met by the learner’s ordering an NSAID as a treatment of myopericarditis
4. **Critical Action 4: Starts peripheral norepinephrine when the patient becomes hypotensive**
  The critical action is met by the learner telling the nurses to start norepinephrine when the BP drops to 80/40 with a MAP of 53
  Cueing Guideline:
  *If the learner orders dobutamine instead of a vasopressor, the patient’s BP will further drop to 65/40 and he will deteriorate into a ventricular fibrillation arrest*
  *If the learner intubates without starting norepinephrine peripherally or clearly states the order of a push dose pressor, then the patient will become unresponsive and will deteriorate into a ventricular fibrillation arrest*
5. **Critical Action 5: Intubates the patient due to persistent hypoxia and drop in GCS but optimizes norepinephrine infusion and considers using push dose pressors**
  The critical action is met by the learner recognizing that the patient is persistently hypoxic with a drop in GCS, increasing the norepinephrine infusion, starting/considering push dose pressors before intubation, and intubating the patient with backup
  Cueing Guideline:
  *If the learner doesn’t recognize a persistent hypoxia and drop in GCS the patient will deteriorate into a ventricular fibrillation arrest*
  *If the learner intubates without starting norepinephrine peripherally or clearly states the order of a push dose pressor, then the patient will become unresponsive and will deteriorate into a ventricular fibrillation arrest*

## ORAL BOARDS ASSESSMENT

*Myopericarditis and Pulmonary Edema*

Learner: _________________________________________

**Critical Actions:**

□ Recognizes a normotensive hypoxic pulmonary edema and connects the patient to an NIV machine
□ Performs a bedside echocardiography and assesses for pericardial effusion and cardiac tamponade
□ Administers aspirin or ibuprofen (NSAID)
□ Starts peripheral norepinephrine when the patient becomes hypotensive
□ Intubates the patient due to persistent hypoxia and drop in GCS but optimizes norepinephrine infusion and considers using push dose pressors

**Summative and formative comments:**

**Milestone assessment:**

## DEBRIEFING AND EVALUATION PEARLS

### Myopericarditis and Pulmonary Edema

Myocarditis and pericarditis are difficult conditions to diagnose in the emergency department (ED) since their presenting complaints are commonly encountered across other disease entities.^2^ They commonly present with chest pain, shortness of breath, and palpitations in otherwise healthy young individuals, often occurring within weeks of a preceding viral infection.^2^ Acute pericarditis is diagnosed when at least two of the following criteria are present: pleuritic or pericarditic chest pain, a pericardial friction rub on physical examination, electrocardiographic changes such as diffuse concave ST-segment elevation with PR-segment depression, or the presence of a pericardial effusion.^1^ Myopericarditis is identified when these findings are accompanied by at least one additional feature, such as elevated cardiac biomarkers, new-onset left ventricular systolic dysfunction on echocardiography or cardiac magnetic resonance imaging (CMR), or evidence of myocardial inflammation on CMR.^1^

The European Society of Cardiology notes that patients with biopsy-confirmed inflammatory heart disease can present with symptoms of acute coronary syndrome (ACS), heart failure, cardiogenic shock, and unstable dysrhythmias.^2^ The patient in this case presented with pulmonary edema and shortness of breath that progressed to cardiogenic shock. In the ED, the initial treatment of pulmonary edema is guided by the patient’s complaints and hemodynamic status. In a normotensive, hypoxic, and tachypneic patient with heart failure, the mainstay of therapy includes non-invasive ventilation (NIV) to promote alveolar clearance, and loop diuretics to promote diuresis.^3^,^5^ High-flow nasal cannula (HFNC) may also be used at flow rates of 40–50 liters per minute; however, it does not generate sufficient positive end-expiratory pressure (PEEP) to adequately promote alveolar fluid clearance.^5^ Also, its use in the context of heart failure has not been adequately studied, and therefore, no strong recommendation can be made regarding its efficacy in this setting.^5^ Otherwise, if NIV is contraindicated or has failed, then endotracheal intubation is indicated.^5^

Acute heart failure and cardiogenic shock should be managed in the standard manner, without disease-specific therapy for myocarditis.^2^ For patients in cardiogenic shock, norepinephrine combined with an inotropic agent such as dobutamine or milrinone is preferred over epinephrine alone.^2^ However, when dobutamine is used alone in cases where the systolic blood pressure is less than or equal to 90mmHg, it may lead to vasodilation and hypotension.^4^ In cases of persistent hypotension or when the patient is in extremis and requires mechanical ventilation, push-dose pressors can be used to increase the blood pressure.^7^,^8^ Epinephrine is a commonly used pressor as push dose because it exhibits both alpha-1 and alpha-2 adrenergic effects as well as beta-1 and beta-2 activity, making it effective.^7^,^8^ Its onset of action occurs in less than one minute, and although a single dose may last up to ten minutes, the hemodynamic effects typically resolve within five minutes. To prepare the push-dose epinephrine, a 10 mL syringe is filled with 9 mL of normal saline and then 1 mL of epinephrine from the cardiac ampule (which contains 10 mL of epinephrine at a concentration of 100 mcg/mL, or 1:10,000) is added into the syringe. The final concentration of epinephrine in the syringe is 10 mcg/mL (1:100,000).^7^,^8^

The mainstay of treatment for pericarditis regardless of the cause is anti-inflammatory drugs combined with supportive care.^2^ The ESC (European Society of Cardiology) guidelines recommend non-steroidal anti-inflammatory drugs (NSAIDs) such as aspirin (650–1000 mg orally every 8 hr) and ibuprofen (600 mg orally every 8 hr) as the first-line therapy in cases of normal renal function.^2^ If there is a contraindication to NSAIDs or failure of treatment, then corticosteroids can be used with a taper regimen.^2^ Colchicine is another first-line therapy recommended to be added to NSAIDs or corticosteroids in acute pericarditis.^1^,^2^ Myocarditis management, though, focuses on supportive care by managing resultant cardiac complications.^2^

Even though the gold-standard test, an endomyocardial biopsy, is not available in the ED, the evaluation of a patient suspected to have myocarditis should include an ECG, troponin, and inflammatory markers, though normal results do not exclude the diagnosis.^1^,^2^ A bedside point-of-care ultrasound (POCUS) should be utilized to detect effusion even though it often reveals normal cardiac function.^1^ While increased pericardial echogenicity has been suggested as a marker of inflammation, it remains a non-specific and limited finding of pericarditis.^1^ In some cases, patients may develop a significant pericardial effusion, which may or may not result in hemodynamic compromise due to tamponade.^1^ Another utility for the bedside ultrasound is to assess volume status in patients with shock.

Patients experiencing refractory cardiogenic shock with hemodynamic instability may require mechanical circulatory support.^2^ Modalities such as intra-aortic balloon pump (IABP), ventricular assist devices (VADs), or extracorporeal membrane oxygenation (ECMO) can serve as a bridge to either ventricular function recovery or cardiac transplantation.^2^ Early initiation within the first 24 hours has been associated with improved outcomes in acute decompensated myocarditis.^2^
